# S100A9 Regulated M1/M2 Macrophage Polarization in Interleukin-10-Induced Promotion of Malignant Pleural Effusion

**DOI:** 10.1155/2023/3473464

**Published:** 2023-07-25

**Authors:** Xue-Bin Pei, Feng-Shuang Yi, Shu-Feng Dong, Qing-Yu Chen, Xin-Yu Shi

**Affiliations:** ^1^Emergency Medicine Clinical Research Center, Beijing Chao-Yang Hospital, Capital Medical University, Beijing, China; ^2^Department of Respiratory and Critical Care Medicine, Beijing Institute of Respiratory Medicine and Beijing Chao-Yang Hospital, Capital Medical University, Beijing, China; ^3^Medical Research Center, Beijing Chao-Yang Hospital, Capital Medical University, Beijing, China

## Abstract

Interleukin-10 (IL-10) promotes the formation and development of malignant pleural effusion (MPE). Previous studies have elucidated the pathogenesis from the view of the immune-regulation function of CD4^+^ T-cells. However, the underlying mechanism is still not fully understood. In this study, our results showed that IL-10 deficiency reduced the percentage of macrophages in mouse MPE and regulated M1/M2 polarization *in vivo* and *in vitro*. The migration capacity of tumor cells was suppressed, and apoptosis was promoted when tumor cells were cocultured with MPE macrophages in the absence of IL-10. Messenger RNA sequencing of MPE macrophages showed that S100A9 was downregulated in IL-10^−/−^ mice. Bone marrow-derived macrophages obtained from wild-type mice transfected with S100A9-specific small interfering RNAs (siRNAs) also showed less M2 and more M1 polarization than those from the siRNA control group. Furthermore, downregulation of S100A9 using S100A9-specific siRNA suppressed MPE development, decreased macrophages, and modulated macrophage polarization in MPE *in vivo*. In conclusion, S100A9 plays a vital role in the process of IL-10 deficiency-mediated MPE suppression by regulating M1/M2 polarization, thus influencing the tumor-migration capacity and apoptosis. This could result in clinically applicable strategies to inhibit the formation of MPE by regulating the polarization of MPE macrophages.

## 1. Introduction

Malignant pleural effusion (MPE) is a common clinical complication that frequently occurs in the setting of advanced malignancy and is associated with high-mortality rates and a short-life expectancy [1, 2]. It is estimated that 15% of patients with lung cancer will have an MPE at presentation [3], and the mean survival time of lung cancer patients with MPEs is 5.5 months, whereas the overall survival time associated with all types of cancer ranges from 3 to 12 months [1]. Several studies have shown that interactions between the tumor cells and host vasculature and immune cells via inflammatory signaling networks are one of the most important aspects leading to the production of an MPE [4–7]. However, the reason why some oncologic patients have MPEs but others do not is still unknown, and the underlying mechanism requires further study to be fully elucidated.

Interleukin-10 (IL-10) is a cytokine with broad anti-inflammatory properties produced by a wide variety of immune cells, including monocytes/macrophages, dendritic cells, B-cells, and T-cell subsets [8, 9]. The biological activities of IL-10 in tumor immunity are extremely controversial and can be highly context-dependent [10, 11]. Some studies indicate that IL-10 positively contributes to tumor growth, whereas others have found that it contributes to the eradication and suppression of angiogenesis and metastasis, which are necessary for longer survival. Research findings also include significantly elevated levels of IL-10 in pleural effusions compared to blood samples, which may imply a specific role for IL-10 in the formation of pleural effusions [12]. Our previous studies demonstrated that IL-10 promoted MPE onset by suppressing the differentiation of T-cells into T_H_1 cells, downregulating the CXCR3/CXCL10 signaling pathway that recruits T_H_1 and T_H_17 cells into MPEs [13], and regulating the T_H_1 response via the miR-7116-5p/GPR55/ERK pathway in mice [14].

It is documented that the important immunosuppressive function of IL-10 involves the suppression of MHC Class II expression and the inhibition of cytokine expression in lipopolysaccharide (LPS)-activated macrophages [15–17]. Also, some *in vivo* studies have reported that macrophages gain a migratory capacity in the absence of IL-10 [18]. In the tumor microenvironment, macrophages are divided into the classical M1 subtype or alternative M2 subtype according to their activation signal. M2 macrophages release immunosuppressive molecules, such as IL-10 and transforming growth factor *β*, to promote tumor growth, while M1 macrophages produce pro-inflammatory cytokines like IL-1*β* and tumor necrosis factor [19, 20]. Previous studies have shown that the macrophages in MPEs exhibit plasticity in being able to transition to M1 or M2 macrophages, and IL-10 could be the key soluble factor in the maintenance of an M2-like state [21]. Here, we report that S100A9 accounts for the regulation of M1/M2 polarization, which plays an important role in IL-10-induced promotion of MPEs.

## 2. Materials and Methods

### 2.1. Cells

Lewis lung cells (LLC, purchased from the American Type Culture Collection ((Manassas, VA))), mouse colon adenocarcinoma (MC38) cells (a gift from Dr. G.T. Stathopoulos of the Department of Physiology, School of Medicine, University of Patras, Rio Patras, Greece) and MC38-GFP cells were cultured at 37°C in 5% CO_2_ using Dulbecco's modified Eagle medium (DMEM) with 10% fetal bovine serum (FBS), penicillin (100 U/mL), streptomycin (100 mg/mL), l-glutamine (2 mM), and pyruvate (1 mM). The detailed information is included in the supporting information.

### 2.2. Mice and MPE Models

MPE models of wild-type (WT) and IL-10^−/−^ mice were prepared by intrapleural injection of 1.5 × 10^5^ MC38 cells or MC38-GFP cells or LLC cells. Ten days after the injection of MC38 cells or 14 days after injection of LLC cells, the mice were sacrificed, and MPE, spleen, and blood samples were collected for experiments. Mononuclear cells were isolated by Ficoll–Hypaque gradient centrifugation (Pharmacia, Uppsala, Sweden), and cell subsets were determined within 1 hr.

For the S100A9 knockdown MPE model, 10 nM of cholesterol-conjugated S100A9 small interfering RNAs (siRNAs) (siS100A9) (Chol-siS100A9) or corresponding controls (Ribobio, Guangzhou, China) was injected intrapleurally every 3 days. The first injection was given at the same time as the tumor injection. At each injection, 40 *μ*L of phosphate buffered saline (PBS)was used to dissolve the siRNA and corresponding vehicle control. The detailed information is included in the supporting information.

### 2.3. Survival Analysis

Mice with MPEs were monitored, euthanized, and recorded as events in the Kaplan–Meier analysis when they were severely sick or moribund. A pairwise log-rank test was used to perform the comparison of overall survival (GraphPad Software, San Diego, CA, USA).

### 2.4. Pleural Permeability Assay

Mice bearing MPEs received 200 *μ*L of 50 mg/mL Evans' blue solution (Sigma–Aldrich, St. Louis, MO, USA) via intravenous injection and were killed 1 hr later. Evans' blue concentrations in the pleural fluid and serum were determined by measuring absorbance at a wavelength of 630 nm compared to the standard Evans' blue concentration.

### 2.5. Production and Stimulation of Bone Marrow-Derived Macrophages (BMDMs)

Mice were sacrificed and bone marrow was extracted to generate BMDMs, as previously described [22]. In brief, bone marrow cells were obtained and filtered through 70 *μ*M nylon mesh to obtain a single-cell suspension. Then, the cells were cultured in a 12-well plate with DMEM supplemented with 10% FBS and recombinant mouse macrophage colony-stimulating factor (20 ng/mL; PeproTech, Cranbury, NJ, USA). After 7 days, differentiated macrophages were washed with PBS and reseeded in a 24-well plate. For macrophage polarization, BMDMs were stimulated with recombinant mouse interferon-*γ* (50 ng/mL) and LPS (100 ng/mL), or recombinant mouse IL-4 (10 ng/mL) for 24 hr to obtain M1 and M2 macrophages, respectively.

### 2.6. Flow Cytometry and Cell Isolation

The antibodies for flow cytometry, including anti-CD11b, anti-F4/80, anti-CD206, and anti-MHC-II monoclonal antibodies, were purchased from Invitrogen (Carlsbad, CA, USA) and BD Biosciences (San Jose, CA, USA). All experiments were analyzed by flow cytometry on a FACS Canto II system (BD Biosciences), and data were examined using the FCS Express 5 software program (De Novo Software, Los Angeles, CA, USA). The flow cytometry methods and strategic steps taken are stated in detail in our previous publications [14, 23]. Tumor cells in MPE (MC38-GFP) can be screened out by flow cytometry. MPE macrophages were isolated using anti-F4/80 microbeads (Miltenyi Biotec, Bergisch Gladbach, Germany). The purity of macrophages was >90%, which was confirmed by flow cytometry, and cells were cultured in DMEM with 10% FBS for further use.

### 2.7. Cell Transfection

BMDMs were transfected with S100A9-specific siRNAs (siS100A9) or negative control (100 nM; RiboBio, Guangzhou, China) using Lipofectamine RNAiMAX reagent (Life Technologies, Carlsbad, CA, USA) according to the manufacturer's recommendations.

### 2.8. RNA Extraction, Reverse Transcription, and qRT–PCR

Total RNA from cells was extracted using TRIzol reagent (Thermo Fisher Scientific, Waltham, MA, USA). RNA was reverse transcribed into complementary DNA for messenger RNA (mRNA) analysis using a reverse transcription kit (Takara Bio, Dalian, China) according to the manufacturer's instructions. The levels of mRNA were quantified by qRT–PCR using SYBR Green I master (Roche, Basel, Switzerland) and determined using the 2^−*ΔΔ*CT^ method in a LightCycler 480 system (Roche). For mRNA detection, glyceraldehyde-3-phosphate dehydrogenase (GAPDH) served as the reference gene. Each sample was assayed in triplicate. The primer sequences are listed in Table S1.

### 2.9. Next-Generation Sequencing

The mRNA expression of macrophages from MPEs of WT or IL-10^−/−^ mice was sequenced using a HiSeq sequencer with a pair-end 150-bp reading length (Illumina, San Diego, CA, USA). A fold-change threshold of two and *P* = 0.05 were used to select significant differentially expressed genes between the two groups. Heatmaps for gene clustering were created using HemI 1.0 [24].

### 2.10. Western Blot

Proteins extracted from cells were separated on 12% sodium dodecyl sulfate–polyacrylamide gel electrophoresis gels and transferred onto nitrocellulose membranes. The blots were blocked with 5% nonfat milk at room temperature for 1 hr and then incubated with specific primary antibodies (S100A9, 1:1000 (Abcam, Cambridge, UK) or GAPDH, 1:6000 (Abcam)) at 4°C overnight, followed by horseradish peroxidase–conjugated secondary antibodies (Abcam) at room temperature for 1 hr. Then, bands were developed with ECL detection reagents (Millipore, Burlington, MA, USA). GAPDH was used as the control.

### 2.11. Transwell Migration Assay

The cell migration assay was performed using 24-well plates with 8.0 *μ*m pore filters (Costar, Cambridge, MA, USA). Tumor cells with a density of 2 × 10^5^ cells/mL were suspended in FBS-free DMEM and seeded in the upper chamber. A total of 2 × 10^5^ macrophages isolated from MPEs of WT and IL-10^−/−^ mice were suspended in DMEM with 10% FBS and added to the lower chamber. After incubation for 24 hr, the migrated cells were fixed in methanol and identified with crystal violet. Cells were photographed with a microscope and counted in each group.

### 2.12. Cell Coculture and Apoptosis Assay

MC38 cells were cocultured with MPE macrophages using a cell culture insert with 0.4 *μ*m pores (Corning, NY, USA). MPE macrophages isolated from WT and IL-10^−/−^ mice were seeded within the upper chamber, while MC38 cells (2 × 10^5^ cells/mL) were plated on the lower chamber. Subsequently, the upper chamber was placed onto the plates. After coculture for 24 hr, MC38 cells were collected, and the apoptotic cells were analyzed using a fluorescein isothiocyanate Annexin V Apoptosis Detection Kit (BD Biosciences).

### 2.13. Statistical Analysis

Data were expressed as mean ± standard deviation. Statistical differences between groups were revealed using Student's *t* test, Student's paired *t* test, or one-way analysis of variance for grouped comparisons, as appropriate. Survival curves were plotted using the Kaplan–Meier method, and differences were evaluated with the log-rank test. Statistical analyses were conducted using GraphPad Prism 7 (GraphPad Software) or SPSS version 19.0 (IBM Corporation, Armonk, NY, USA), and *P* < 0.05 was considered statistically significant.

## 3. Results

### 3.1. IL-10 Deficiency Suppressed MPE Production and Decreased Macrophages in MPEs

**Figure 1:**
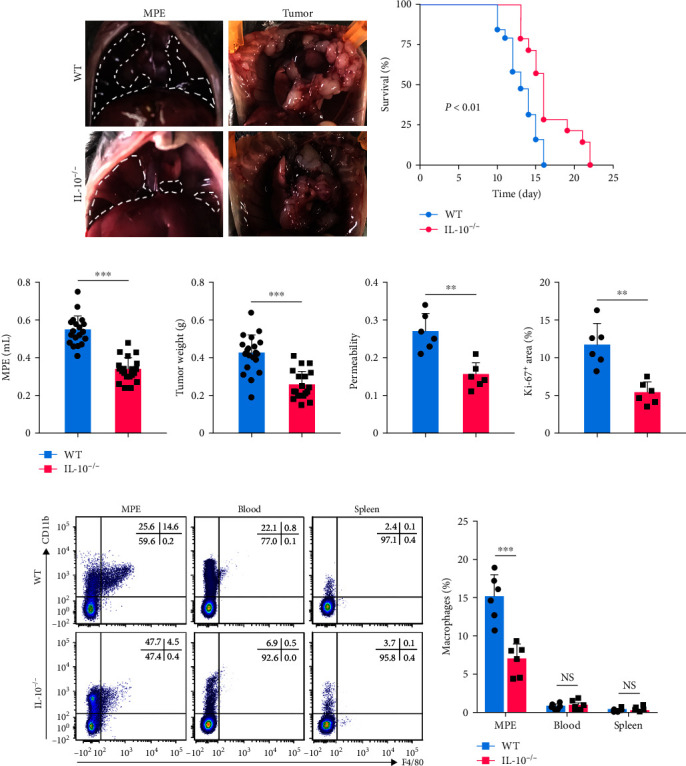
IL-10 deficiency suppresses the formation of MPE and decreases total macrophages in MPE. (a) Anatomical images of pleural effusion and intrapleural tumors of WT and IL-10^−/−^ mice. (b) Life span analysis was performed in mice receiving intrapleural injections of MC38 cells. The overall survival of the two groups was analyzed using the Kaplan–Meier method and a pairwise log-rank test (each *n* = 20). (c) Comparisons of MPE volume (each *n* = 20), pleural tumor mass (each *n* = 20), pleural vascular permeability (represented as the MPE/serum ratio of Evans blue concentration, each *n* = 6), and Ki67^+^ area in pleural tumors (each *n* = 6) between WT and IL-10^−/−^ mice. (d) Representative flow cytometric dot plots (left panel) and statistical comparisons (right panel) of macrophages (CD11b^+^F4/80^+^) in MPEs, blood, and spleens of WT and IL-10^−/−^ mice. Data are presented as mean ± standard deviation (SD).  ^*∗*^*P* < 0.05,  ^*∗∗*^*P* < 0.01,  ^*∗∗∗*^*P* < 0.001, compared by Student's *t* test.

MC38 cells were intrapleurally injected into IL-10^−/−^ and WT mice; then, 10 days later, MPEs and pleural tumors were collected and analyzed. Our results showed that IL-10 deficiency suppressed the production of MPE and tumors (Figure 1(a)), and prolonged the survival of mice bearing MPEs (Figure 1(b)). Significant declines in MPE volume and tumor mass were observed in the IL-10^−/−^ group compared to the WT control group (Figure 1(c), left two panels). The Evan's blue MPE/blood ratio and Ki67^+^ area in pleural tumors were decreased in the IL-10^−/−^ group (Figure 1(c), right two panels), indicating that IL-10 absence suppressed the development of MPEs by reducing vascular permeability and inhibiting tumor angiogenesis and proliferation, consistent with the findings of previous studies [13, 14]. We further tested the profiles of immune cells. As shown in Figures 1(d) and Figure S1(a), the percentage of macrophages in the MPEs of IL-10^−/−^ mice was significantly lower than that in the MPEs of WT mice.

### 3.2. IL-10 Regulated M1/M2 Polarization In Vivo and In Vitro

**Figure 2:**
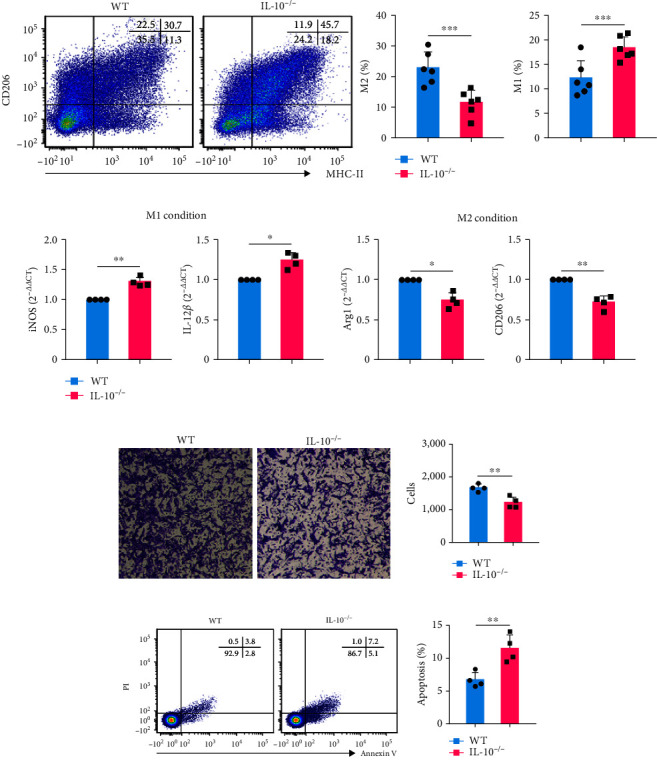
IL-10 regulates M1/M2 macrophage polarization in MPEs. (a) Representative flow cytometric dot plots (left panel) and statistical comparisons (right panel) of M2 macrophages (CD206^+^MHC-II^−^) and M1 macrophages (CD206^−^MHC-II^+^) in MPEs of WT and IL-10^−/−^ mice (*n* = 6). (b) Comparisons of mRNA expression of iNOS, IL-12*β*, Arg-1, and CD206 in WT and IL-10^−/−^ BMDMs activated under the M1 or M2 condition (each *n* = 4). (c) Migration capacity of MC38 cells cocultured with WT and IL-10^−/−^ mouse MPE macrophages was analyzed *in vitro* using a transwell coculture system; the representative images (left panel) and statistically significant numbers of migrated cells (right panel) are shown (*n* = 4). (d) MC38 cells were cocultured with macrophages from MPE, and the apoptosis of MC38 cells was evaluated. Representative flow cytometric dot plots (left panel) and comparisons (right panel) of the apoptotic fraction of MC38 cells cocultured with macrophages from MPEs of WT and IL-10^−/−^ mice (*n* = 4). Data are presented as mean ± SD.  ^*∗*^*P* < 0.05,  ^*∗∗*^*P* < 0.01,  ^*∗∗∗*^*P* < 0.001, compared by Student's *t* test.

Macrophages are commonly classified as either M1 or M2 phenotype, although the phenotypes and populations of macrophages are heterogeneous [25]. In lung tissues, macrophages show altered phenotypes and functions [26]. To investigate the phenotypes and functions of macrophages in MPEs, we first tested the M1/M2 spectrum in MPEs of WT and IL-10^−/−^ mice. The results (Figures 2(a) and Figure S1(b)) showed that M2 macrophages (CD206^+^MHC-II^−^) were decreased and M1 macrophages (CD206^−^MHC-II^+^) were increased in IL-10^−/−^ group, and the difference was statistically significant. To figure out how IL-10 regulates M1/M2 polarization in MPEs, BMDMs of WT, and IL-10^−/−^ mice were isolated and cultured under the M1 condition (interferon-*γ*, 50 ng/mL and LPS, 100 ng/mL) or M2 condition (IL-4, 10 ng/mL), respectively. The expression levels of iNOS and IL-12*β* were significantly higher in the IL-10^−/−^ group under the M1 condition compared to M2 condition (Figure 2(b), left panel). In the M2 condition, the levels of Arg-1 and CD206 were lower in the IL-10^−/−^ group than in the WT group. The results showed that IL-10 regulates M1/M2 polarization *in vitro* and *in vivo*, which might play an important role in influencing the production and development of MPEs.

To further research whether IL-10 affects the function of MPE macrophages, MC38 cells were cocultured with MPE macrophages from WT and IL-10^−/−^ mice *in vitro*. In the transwell system, MPE macrophages from WT mice significantly promoted more MC38 cell migration compared to those from the IL-10^−/−^ group, as shown in Figure 2(c). Besides, the effects of macrophages on MC38 cell apoptosis were also analyzed.  Figure 2(d) shows that macrophages from WT mice inhibited MC38 cell apoptosis more significantly compared to those from the IL-10^−/−^ group.

### 3.3. Downregulation of S100A9 Was Related to M1/M2 Polarization In Vitro

**Figure 3:**
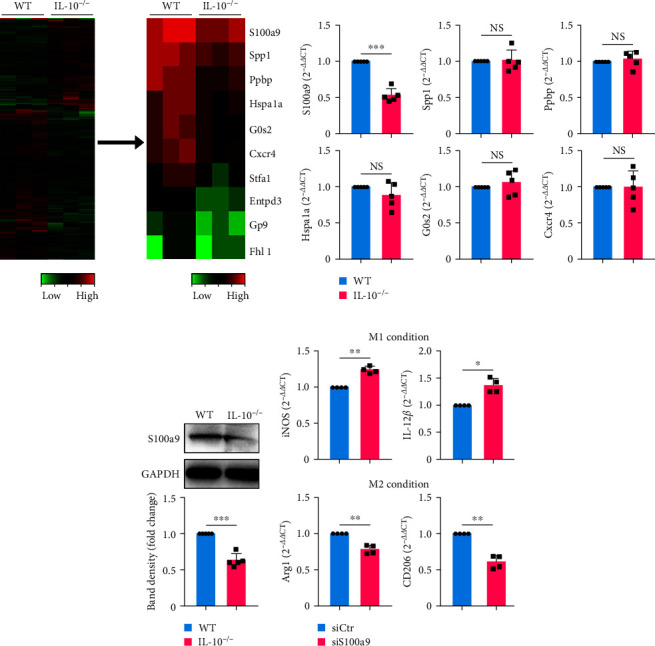
Downregulation of S100A9 expression is associated with M1/M2 macrophage polarization *in vitro*. (a) Heatmap shows a differential expression of mRNAs in MPE macrophages between WT and IL-10^−/−^ mice. The red color indicates a relatively high expression, and the green color indicates a relatively low expression. (b) Comparison of mRNA expression of S100A9, Spp1, Ppbp, Hspa1a, G0s2, and Cxcr4 in MPE macrophages from WT and IL-10^−/−^ mice (verified by quantitative RT–PCR, each *n* = 5). (c) Expression of S100A9 was analyzed by Western blot in MPE macrophages from WT and IL-10^−/−^ mice. Band densities of samples were analyzed and expressed as a fold change of normalized results from corresponding control cells (*n* = 5). (d) Comparison of mRNA expression of iNOS, IL-12*β*, Arg-1, and CD206 in WT BMDMs activated under the M1 or M2 condition in the presence of siCtr or siS100A9. Data are presented as mean ± SD.  ^*∗*^*P* < 0.05,  ^*∗∗*^*P* < 0.01,  ^*∗∗∗*^*P* < 0.001, compared by Student's *t* test.

To gain further insight into the IL-10-induced M1/M2 polarization, macrophages were isolated from MPEs of WT and IL-10^−/−^ mice, and total RNA was extracted and subjected to mRNA sequencing (GSE220281). Significant differential expression of genes was characterized as a clustered heatmap (Figure 3(a), left panel), and representative differential genes were listed (see Figure 3(a), right panel). After quantitative RT–PCR and Western blot confirmation, S100A9 expression was confirmed to be downregulated in IL-10^−/−^ MPE macrophages (Figures 3(b) and 3(c)). To elucidate whether S100A9 downregulation was associated with M1/M2 polarization, siS100A9 was designed and transfected into BMDMs separated from WT mice *in vitro*. As shown in Figure 3(d), siS100A9 treatment inhibited M2 polarization and promoted M1 polarization.

### 3.4. Downregulation of S100A9 Expression Contributed to MPE Suppression by Regulating M1/M2 Polarization

**Figure 4:**
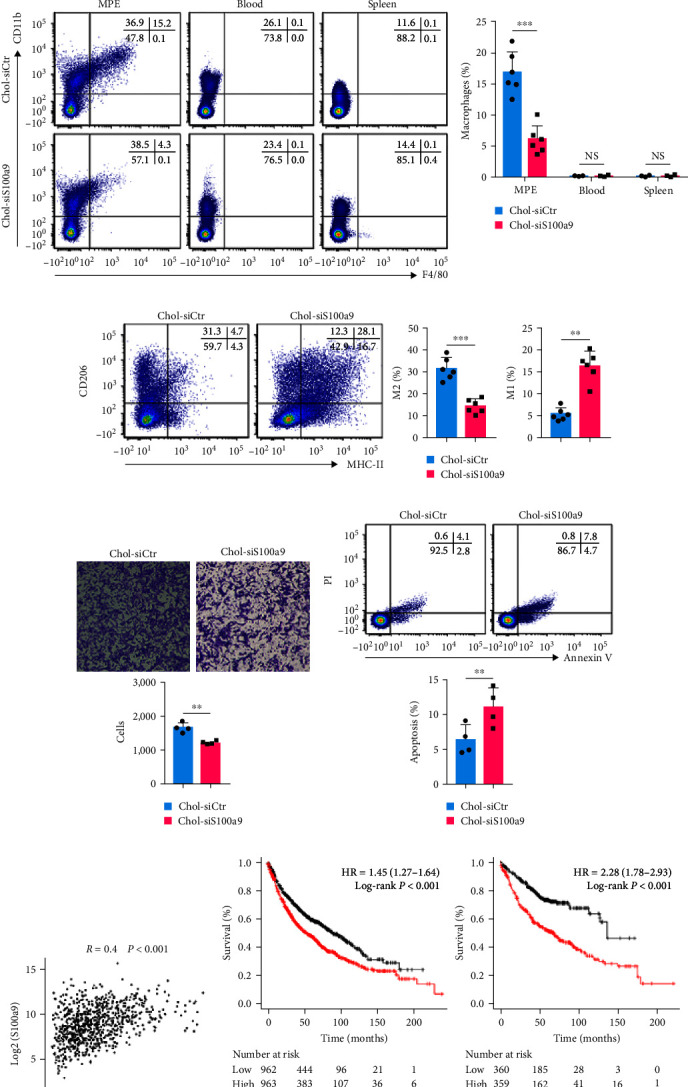
S100A9 knockdown in mice suppressed the development of MPE and regulated M1/M2 macrophage polarization in MPE. (a) S100A9 was knocked down by the application of Chol-siS100A9 in WT mice, and the downregulation of S100A9 in MPEs, blood, and spleens was verified by qRT–PCR (each *n* = 5). (b) Comparisons of MPE volume and intrapleural tumors in mice receiving Chol-siS100A9 and cholesterol-conjugated siCtr (Chol-siCtr). (c) Representative flow cytometric dot plots (left panel) and statistical comparisons (right panel) of MPE macrophages of the S100A9-knockdown and control groups (each *n* = 6). (d) Representative flow cytometric dot plots (left panel) and statistical comparisons (right panel) of M2 and M1 macrophages in MPEs from the S100A9-knockdown and control groups (each *n* = 6). (e) Transwell migration assay was performed on MC38 cells treated with macrophages from MPEs of the S100A9-knockdown and control groups; the representative images (upper panel) and statistically significant numbers of migrated cells (lower panel) are shown (*n* = 4). (f) Representative flow cytometric dot plots (upper panel) and comparisons (lower panel) of apoptosis in MC38 cells cocultured with macrophages from MPEs of the S100A9-knockdown and control groups (*n* = 4). (g) Correlation analysis of IL-10 and S100A9 gene expression was performed by GEPIA (data were from Kaplan–Meier plotter, http://gepia.cancer-pku.cn/index.html). (h) Overall survival of ungrouped lung cancer patients (left panel) and lung adenocarcinoma patients (right panel) with high- or low-S100A9 expression was evaluated using the Kaplan–Meier survival curves and compared using the pairwise log-rank tests (data were from Kaplan–Meier plotter, http://kmplot.com/analysis/). Data are presented as mean ± SD.  ^*∗*^*P* < 0.05,  ^*∗∗*^*P* < 0.01,  ^*∗∗∗*^*P* < 0.001, compared by Student's *t* test.

To further characterize the effects of S100A9 on MPE suppression, we inhibited S100A9 expression *in vivo* (Chol-siS100A9, modified with cholesterol, based on siS100A9 *in vitro*). The results showed that Chol-siS100A9 inhibited S100A9 expression in macrophages from MPEs, blood, and spleens compared to the cholesterol-conjugated control (Chol-siCtr) (Figure 4(a)). MPE volume and tumor weight were significantly decreased in the Chol-siS100A9 group (Figure 4(b)).

The percentage of macrophages in MPE was significantly decreased in MPEs when S100A9 was suppressed (Figure 4(c)). We examined the M1/M2 spectrum in MPE, and consistent with the *in vitro* results, Chol-siS100A9 administration also decreased M2 macrophages and increased M1 macrophages in treated MPEs compared to those in the control group (Figure 4(d)). After treatment with Chol-siS100A9 or the corresponding control, macrophages were separated from MPEs for further study. After coculturing with macrophages from MPEs, the migration capacity of MC38 cells was inhibited (Figure 4(e)) and apoptosis was promoted (Figure 4(f)) in Chol-siS100A9 mice compared with those in the control group.

The correlation between IL-10 and S100A9 gene expression in lung cancer was analyzed using the Gene Expression Profiling Interactive Analysis database, and it showed a positive correlation between IL-10 and S100A9 (*R* = 0.4, *P* < 0.001) (Figure 4(g)). In addition, we explored the potential relationship between the expression levels of S100A9 and human lung cancer progression. It was noted that a greater expression of S100A9 was associated with a lower overall survival rate both in patients with ungrouped lung cancer and patients with lung adenocarcinoma (Figure 4(h)).

## 4. Discussion

MPE is a common and challenging problem worldwide due to its high morbidity, mortality, and associated health care cost and burden. More than 1 million people globally are diagnosed with MPEs each year [27]. Moreover, the incidence of MPE onset is likely to increase as cancer rates rise worldwide. A great deal of research work has been done to elucidate the mechanism of MPEs. Mounting evidence indicates that the interactions between cancer cells and immune cells, as well as the vascular leakiness leading to fluid accumulation in the pleural space, are mainly responsible for MPE formation [28, 29]. Our previous data showed that IL-10 promotes the development of MPEs in mice by regulating the differentiation and migration of T_H_1 and T_H_17 cells [13], and IL-10 inhibits the expansion of T_H_1 cells via the miR-7116-5p/GPR55/ERK signaling pathway, resulting in MPE aggravation [14]. In this study, we extended our previous findings and revealed that IL-10 induces the production of MPE via S100A9-mediated M1/M2 polarization.

IL-10 plays a key role in tumor immune evasion as well as its immunomodulatory functions with both immunosuppressive and immunostimulatory activities. It is essential to understand the regulatory mechanism of IL-10 in MPE formation, both for deciphering how IL-10 acts in regulating tumor immunity and for discovering important molecular targets for intervention in cancers. Our previous studies on the role of IL-10 in MPEs mostly focused on the function of T-cells, especially CD4^+^ T-cells, but the pathogenesis of IL-10-induced MPE has not been fully elucidated. Cardoso et al. [30] reported that IL-10-stimulated macrophages are more effective than LPS-stimulated macrophages with a pro-inflammatory effect at promoting the invasion of gastric and colorectal cancer cells. In the microenvironment of MPE, IL-10 may play an important role in the formation of the MPE by affecting the function and spectrum of macrophages; thus, we have focused on elucidating this issue from the viewpoint of M1/M2 macrophage activation.

Depending on different activation signals, macrophages could be divided into the classical M1 type or an alternative M2 type in the tumor microenvironment. It has been reported that immune escape in the tumor microenvironment leads to the repolarization of macrophages from M1 to M2 in the late stage of tumor progression and metastasis [31]. Our results showed that, in the MPE microenvironment, the percentage of M2 macrophages showed a significant decrease in the absence of IL-10. *In vitro*, BMDMs isolated from WT and IL-10^−/−^ mice showed similar results as MPE macrophages when activated in the M1 or M2 condition, respectively. The functions of macrophages are affected by the surrounding environments and vary with their phenotype transition [32]. During this study, we further investigated the changes in macrophage function. We also observed that IL-10 deficiency suppressed the migration capacity of tumor cells and promoted tumor cell apoptosis, indicating that tumor cells with impaired migration, and viability contribute to pleural stomata occlusion and lymph vessel obstruction to a lesser extent.

It has been demonstrated that MPE macrophages of lung adenocarcinoma cancer patients within the unique M1/M2 spectrum show plasticity in the M1–M2 transition [21]. To address the role of IL-10 in the regulation of M1/M2 polarization in MPE, we compared mRNA expression profiles between MPE macrophages derived from WT and IL-10^−/−^ mice, and S100A9 was confirmed to be downregulated in IL-10^−/−^ mice. It has been reported that S100A9 is expressed in a wide variety of cell types and is abundant in neutrophils, monocytes, and keratinocytes and in the early differentiation states of macrophages [33, 34].

Previous studies found that elevated levels of S100A8/S100A9 (a member of the Ca^2+^ binding protein of the S100 family) were present in inflammation and various cancers in humans, and these observations have triggered widespread concern about S100A8/S100A9 as a new potential molecule with important roles in the process of immune modulation, which promotes malignancy formation and progression [35]. Several studies have reported that S100A9 participates in the process of development of various diseases by influencing the polarization and function of macrophage subtypes; however, the results vary across different disease models. Ganta et al. [36] reported that S100A8/A9 induces M1 macrophage phenotype polarization in ischemic macrophages in peripheral artery disease. S100A9 attenuates the development of M2 and induces pro-inflammatory functions in obesity [37]. Under hyperglycemic conditions, the upregulation of S100A9 induces an activating histone code on the gene promoter in M1 macrophages [38]. S100A9 treatment promotes BMDMs to evolve from M0 to M2 polarization, maintaining the immunosuppression function of testicular macrophages [39]. We reveal a yet-unrecognized impact of S100A9 on M1/M2 polarization, in that it contributes to an impaired tumor-migration capacity and increased apoptosis and subsequent longer survival period among patients with MPEs.

Overall, the downregulation of S100A9 plays a vital role in the process of IL-10 deficiency-mediated MPE suppression by the regulation of M1/M2 polarization, influencing tumor migration capacity, and apoptosis. This could lead to clinically applicable strategies to inhibit MPE formation by regulating the polarization of MPE macrophages. [1]

## Data Availability

The research data used to support the findings of this study are included within the article.

## Supplementary Materials

Supplementary Materials Table S1: primer sequences. Figure S1: IL-10 deficiency decreases total macrophages in MPE and IL-10 regulates M1/M2 macrophage polarization in MPEs in LLC models.
