# Supplementary material for: S100A9 Regulated M1/M2 Macrophage Polarization in Interleukin-10-Induced Promotion of Malignant Pleural Effusion
Source: J Immunol Res. 2023 Jul 25;2023:3473464. doi: 10.1155/2023/3473464 (PMC10393522; doi:10.1155/2023/3473464)
Supplement: Supplementary Materials — Table S1: primer sequences. Figure S1: IL-10 deficiency decreases total macrophages in MPE and IL-10 regulates M1/M2 macrophage polarization in MPEs in LLC models. [file 3473464.f1.docx]

**Details of Materials and Methods**

**Cells**

To obtain MC38-GFP cells, MC38 cells were transfected with the lentivirus with GFP fragment plus polybrene for 48 hours, positive cells were collected by flow cytometry. MC38-GFP cells with a purity > 96% were used in experiments.

**Mice and MPE models**

WT C57BL/6 mice were purchased from Beijing Vital River Laboratory Animal Technology (Beijing, China). *IL-10^–/–^*mice with a C57BL/6 background (Stock No. 002251) were purchased from Jackson Laboratory (Bar Harbor, ME). Mice used for experiments were sex, weight (20–25 g), and age (6–8 wk) matched. The mice were bred on a chow diet in a 12-hour: 12-hour light-dark environment at 25 °C in the Animal Center of Capital Medical University under high health status conditions. The animal study protocol was approved by the Institutional Animal Care and Utilization Committee of Capital Medical University.

Mouse models of MPE were prepared by intrapleural injection of 1.5 × 10^5^ LLC, MC38, or MC38-GFP cells as described previously. The cell pellets of MPE, blood, and spleen were resuspended in PBS, and mononuclear cells were isolated by Ficoll-Hypaque gradient centrifugation (Pharmacia, Uppsala, Sweden) within 1 hour to determine T-cell subsets. All the animal studies were approved by the Institutional Animal Care and Utilization Committee of Capital Medical University.

Cholesterol-conjugated S100A9-specifific small interfering RNAs (siRNAs) (Chol-siS100A9) and their corresponding negative controls (Chol-siCtr, from RiboBio, Guangzhou, China) were injected intrapleurally for siRNA transfection in vivo. Ten nanomole of Chol-siS100A9 or Chol-siCtr dissolved in 0.04 mL saline buffer was locally injected into the pleural cavity once every 3 d during MPE formation of murine models.

**Supplemental Figure Legend**

**
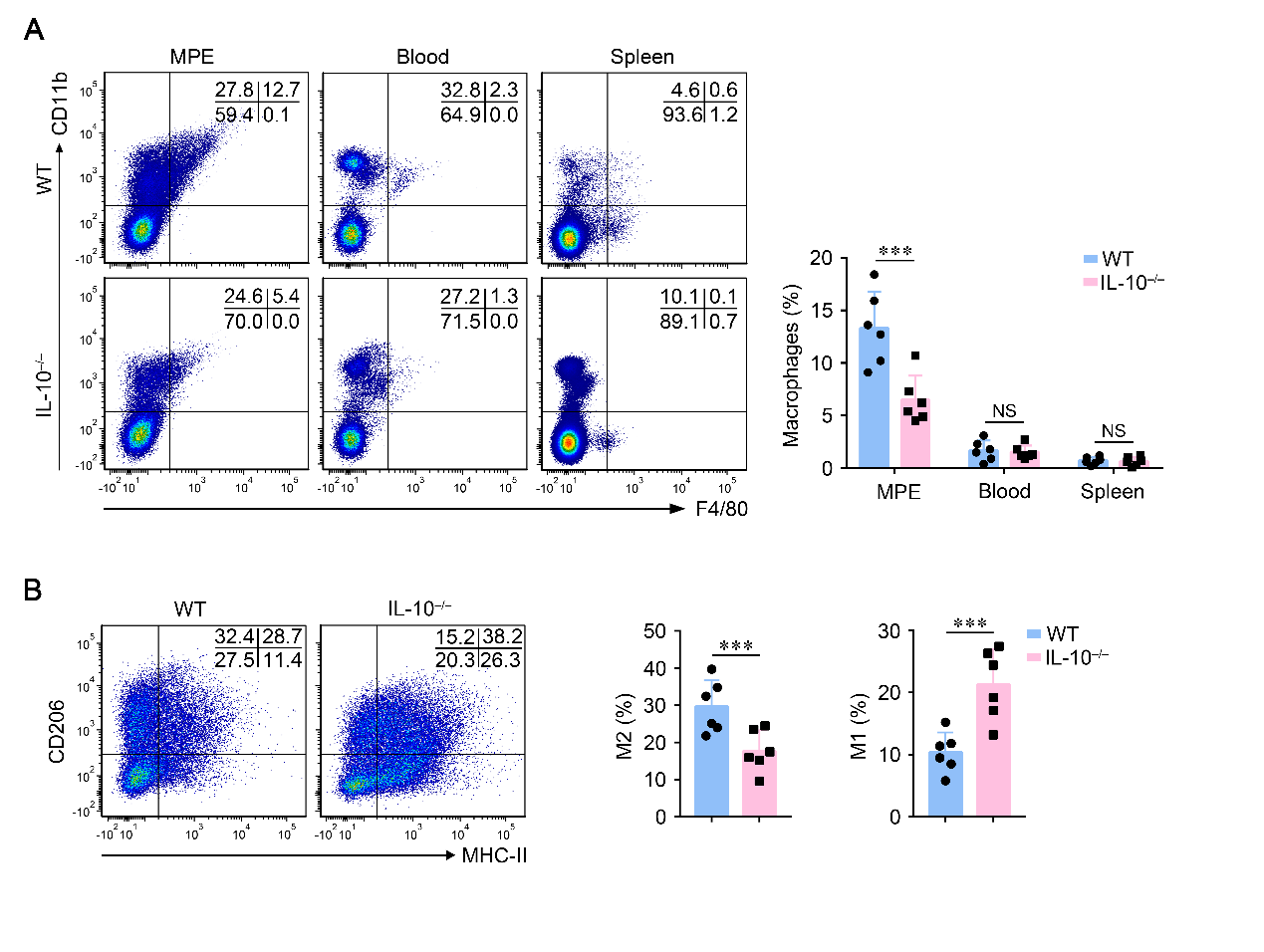
**

Supplemental Figure 1. IL-10 deficiency decreases total macrophages in MPE and IL-10 regulates M1/M2 macrophage polarization in MPEs in LLC models. (A) Representative flow cytometric dot plots (left panel) and statistical comparisons (right panel) of macrophages (CD11b^+^F4/80^+^) in MPEs, blood, and spleens of WT and *IL-10*^−/−^ mice in LLC models. (B) Representative flow cytometric dot plots (left panel) and statistical comparisons (right panel) of M2 macrophages (CD206^+^MHC-II^-^) and M1 macrophages (CD206^-^MHC-II^+^) in MPEs of WT and *IL-10*^−/−^ mice (n = 6). Data are presented as mean ± SD. **P <* 0.05, ***P* < 0.01, ****P* < 0.001, compared by Student’s *t* test.

**Supplemental Table 1 │ Primer sequences**

| Gapdh Forward | TGGATTTGGACGCATTGGTC |
| --- | --- |
| Gapdh Reverse | TTTGCACTGGTACGTGTTGAT |
| Inos Forward | GTTCTCAGCCCAACAATACAAGA |
| Inos Reverse | GTGGACGGGTCGATGTCAC |
| IL-12β Forward | TGGTTTGCCATCGTTTTGCTG |
| IL-12β Reverse | ACAGGTGAGGTTCACTGTTTCT |
| Arg1 Forward | CTCCAAGCCAAAGTCCTTAGAG |
| Arg1 Reverse | AGGAGCTGTCATTAGGGACATC |
| CD206 Forward | CTCTGTTCAGCTATTGGACGC |
| CD206 Reverse | CGGAATTTCTGGGATTCAGCTTC |
| S100a9 Forward | ATACTCTAGGAAGGAAGGACACC |
| S100a9 Reverse | TCCATGATGTCATTTATGAGGGC |
| Spp1 Forward | AGCAAGAAACTCTTCCAAGCAA |
| Spp1 Reverse | GTGAGATTCGTCAGATTCATCCG |
| Ppbp Forward | CTCAGACCTACATCGTCCTGC |
| Ppbp Reverse | GTGGCTATCACTTCCACATCAG |
| Hspa1a Forward | TGGTGCAGTCCGACATGAAG |
| Hspa1a Reverse | GCTGAGAGTCGTTGAAGTAGGC |
| G0s2 Forward | GTGAAGCTATACGTGCTGGG |
| G0s2 Reverse | CCGTCTCAACTAGGCCGAG |
| Cxcr4 Forward | GACTGGCATAGTCGGCAATG |
| Cxcr4 Reverse | AGAAGGGGAGTGTGATGACAAA |
| Entpd3 Forward | TTGTGAGCATTGTGGTACTTGT |
| Entpd3 Reverse | GGCCACTGATACACGTAGACAG |
| Gp9 Forward | TGCGACCACAGATACTCAGG |
| Gp9 Reverse | ACTGAACGCAGGCTATTGTTG |
| Fhl1 Forward | GACTGCCGCAAGCCCATAA |
| Fhl1 Reverse | CCAAGGGGTGAAGGCACTT |
